# Fluoxetine as a Potential Therapeutic Agent for Inhibiting Melanoma Brain and Lung Metastasis: Induction of Apoptosis, G0/G1 Cell Cycle Arrest, and Disruption of Autophagy Flux

**DOI:** 10.7150/jca.95592

**Published:** 2024-05-20

**Authors:** Anqi He, Mengling Wu, Yamin Pu, Ru Li, Yiwen Zhang, Jing He, Yong Xia, Yimei Ma

**Affiliations:** 1 Department of Rehabilitation Medicine and Institute of Rehabilitation Medicine, West China Hospital, Sichuan University, Chengdu, 610041, China.; 2 Department of Pediatrics, West China Second University Hospital, Sichuan University, Chengdu, China.; 3 Key Laboratory of Birth Defects and Related Diseases of Women and Children (Sichuan University), Ministry of Education, Chengdu, China.; 4 Key Laboratory of Rehabilitation Medicine in Sichuan Province, West China Hospital, Sichuan University, Chengdu, 610041, China.; 5 Department of Biotherapy, Cancer Center and State Key Laboratory of Biotherapy, West China Hospital, Sichuan University, Chengdu, 610041, China.; 6 Innovation Center of Nursing Research, Nursing Key Laboratory of Sichuan Province, West China Hospital, Sichuan University /West China School of Nursing, Sichuan University, Chengdu, 610041, China.

**Keywords:** Melanoma metastasis, Apoptosis, Autophagy, Cell cycle arrest

## Abstract

Brain metastases and lung metastases are major causes of treatment failure and related mortality in melanoma. Fluoxetine hydrochloride (FXT), a widely-used antidepressant, has emerged as a potential anticancer agent in preclinical studies. Previous research has shown its potential to inhibit melanoma. However, its efficacy and the underlying mechanisms in melanoma metastasis, especially concerning brain metastases and lung metastases, remain underexplored. This study investigates FXT's inhibitory effects on melanoma growth and metastasis to the lung and brain. Employing a combination of in vitro assays, we demonstrate FXT's potent suppression of melanoma growth through induction of intrinsic apoptosis, disruption of autophagic flux, and cell cycle arrest at the G0/G1 phase. In in vivo mouse models, we found that FXT exhibits strong inhibitory activity against melanoma brain metastases and lung metastases. Our findings provide a foundation for future clinical exploration of FXT as a novel treatment strategy for melanoma, underscoring its ability to target both primary and metastatic lesions.

## 1. Introduction

Melanoma is a type of malignant skin tumor characterized by high invasiveness and metastatic potential, posing a significant threat to human health 1. The incidence of melanoma has been steadily increasing over the past few decades, particularly among Caucasians 2. Various factors contribute to the development of melanoma, including genetics 3, prolonged exposure to ultraviolet radiation 4, and abnormal immune system function 5. Excessive exposure to ultraviolet radiation is considered the primary risk factor 4.

Early-stage melanoma can be completely cured through surgical removal, including the excision of the primary tumor and potential lymph node metastases 6. For advanced-stage melanoma, immunotherapy and targeted therapy are important treatment strategies 7, 8. Immunotherapy utilizes immune checkpoint inhibitors to enhance the immune system's response to the tumor 7. Targeted therapy focuses on specific gene mutations in melanoma and utilizes targeted drugs to inhibit the action of these mutated genes 8. However, melanoma treatment currently faces several challenges. Some melanoma patients are insensitive to conventional treatment methods or develop resistance, which limits the durability of treatment effectiveness 9.

Furthermore, melanoma often exhibits distant metastasis during the advanced stages of its progression 10. Among them, brain metastasis and lung metastasis are among the most common pathways of melanoma metastasis 11. Studies have shown that up to 60% of melanoma patients develop brain metastasis during the course of disease progression 12. This type of metastasis often leads to the appearance of neurological symptoms such as confusion, abnormal sensations, hemiparesis, increased intracranial pressure, meningeal signs, seizures, and aphasia 13. Strategies for treating brain metastasis encompass surgical removal, stereotactic radiosurgery, and whole-brain radiation therapy 14. In recent years, targeted therapy and immunotherapy for brain metastasis have also made some progress 15. However, the treatment of brain metastasis remains challenging due to the unique properties of brain tissue, such as the presence of the BBB and the multiplicity of tumors, which make treatment efficacy and prognosis more complex. Additionally, lung metastasis is also very common in melanoma patients 16. Lung metastasis can cause respiratory-related symptoms such as difficulty breathing, coughing, and chest pain 17. Treatment strategies for lung metastasis primarily include surgical resection, radiation therapy, and systemic therapy 18. In recent years, immunotherapy has shown potential in the treatment of melanoma lung metastasis, particularly the use of immune checkpoint inhibitors 7. However, the treatment of lung metastasis also faces challenges, such as the number and distribution of lung metastases, treatment resistance, which can affect treatment efficacy and prognosis.

Brain metastasis and lung metastasis have a significant impact on the prognosis and treatment strategies for melanoma patients. Therefore, the search for more effective treatment methods and improved prognosis for melanoma brain metastasis and lung metastasis is an urgent problem that needs to be addressed.

Repurposing old drugs is an economical and efficient drug development strategy that can expedite the process of developing new drugs. FXT is a selective serotonin reuptake inhibitor commonly used in the treatment of depression 19. In recent years, researchers have discovered its potential anti-tumor effects 20, 21. Studies have shown that FXT can inhibit the proliferation of various tumor cell lines, including breast cancer 21, liver cancer, and lung cancer 20. This inhibitory effect may be associated with FXT's regulation of the cell cycle 22, induction of apoptosis 23, and inhibition of cell growth signaling pathways 24. Furthermore, FXT not only inhibits tumor cell proliferation but also demonstrates potential anti-metastatic properties 25. Research has found that FXT can suppress the invasive and migratory abilities of tumor cells, as well as inhibit the process of angiogenesis, thereby blocking tumor metastasis 25. FXT demonstrates its anti-tumor effects by influencing various signaling pathways linked to tumor development 24. For instance, FXT can inhibit the Wnt/β-catenin signaling pathway 26, the PI3K/Akt signaling pathway 27, and the NF-κB signaling pathway 28, thereby suppressing tumor cell proliferation and metastasis. However, despite showing certain therapeutic efficacy in the treatment of various tumors, further clinical trials and research are still needed to validate its safety and effectiveness.

Considering its anti-proliferative and anti-metastatic effects and its ability to modulate tumor-related signaling pathways, FXT has garnered attention as a potential therapeutic option for melanoma brain and lung metastasis. However, the specific activity and mechanisms of FXT in treating melanoma, particularly regarding BBB penetration, require further investigation. This study aims to explore the inhibitory activity and mechanisms of FXT in melanoma and its brain metastasis, offering new strategies and potential drug candidates for the treatment of this aggressive tumor.

## 2. Materials and Methods

### 2.1 Cell lines

The cell lines A375, A875, A2058, C32, WM115, HT144, B16-F10, MDA-MB-231, 4T1, CT26, HDF-a, L929, NCM460 were procured from the U.S. Type Culture Collection (ATCC) within the last 5 years. Within the past 3 years, STR analysis has authenticated the human cell lines, confirming the absence of mycoplasma in all cell lines.

### 2.2 Cell viability assay

A cell viability assay, specifically the MTT assay (3-(4,5-dimethylthiazol-2-yl)-2,5-diphenyltetrazolium bromide), was employed to evaluate the number or percentage of viable cells within a cell population. For this assay, 1.0-1.5×10^3^ cells were seeded in 96-well plates and exposed to varying concentrations of FXT. Subsequently, these cells were treated with 20 µL of 5 mg/mL MTT solution and incubated for 2-3 hours at 37°C following 24-hour, 48-hour, and 72-hour drug exposures. Post-incubation, 150 µL of DMSO was added to each well after removing the medium. During this assay, viable cells' mitochondrial enzymes convert the yellow MTT reagent into a purple formazan product. Spectrophotometric quantification of this formazan product at 562 nm was performed using a microplate spectrophotometer (Molecular Devices, CA, USA), offering an indirect measure of cell viability. The IC50 values were determined using GraphPad Prism 8 software.

### 2.3 Colony formation assay

The colony formation assay is a commonly used technique to assess the clonogenic potential of cells and their ability to form colonies. It is often employed to evaluate the long-term effects of treatments or substances on cell proliferation and survival. Cells are seeded into 6-well plates at a low density to ensure that individual cells have enough space to proliferate and form colonies. After allowing the cells to adhere to the plate, they are treated with FXT (the specific treatment of interest) for a period of 7-10 days. The cells are incubated under appropriate conditions (temperature, humidity, and CO2 levels) to promote cell growth and colony formation. Then Using a microscope or imaging system, the stained colonies are observed and counted. A colony is typically defined as a cluster of at least 50 cells.

### 2.4 Cell cycle and apoptosis analysis

Melanoma cells were treated with FXT for 12h, 24h or 48h, stained with propidium iodide (PI) for 15-60 min to ensure optimal staining and DNA intercalation. After the treatment period, the cells are harvested by detaching them from the culture dish or plate using a suitable enzyme or gentle cell dissociation method. Stained cells are analyzed using flow cytometry. By measuring the intensity of PI fluorescence, the DNA content of individual cells can be determined. The resulting data provide information about the distribution of cells in different phases of the cell cycle (G0/G1, S, G2/M), allowing the assessment of changes induced by the FXT treatment. Apoptosis analysis was performed using Annexin V-PE/7-AAD staining, followed by FCM analysis.

### 2.5 Mitochondrial membrane potential (ΔΨm) and reactive oxygen species (ROS) measurements

Cells were treated with FXT for 48 hours, stained with 2',7'-dichlorofluorescin diacetate (DCFH-DA) (10 µM) or rhodamine 123 (Rh123) (5 µg/ mL) for 30 min, then analyzed using FCM to measure the levels of ROS and ΔΨm levels, respectively. Each assay was repeated three times.

### 2.6 Western blotting

Protein extraction was performed from cells treated with FXT for 48 h. Specific primary and secondary antibodies were used for Western blot analysis. The proteins transferred onto membranes were visualized using a ChemiScope 6200 Touch chemiluminescence imaging system, and the results were quantified using ImageJ software.

### 2.7 Plasmid transfection and immunofluorescence analysis

GFP-RFP-LC3 plasmids were transfected into A375 cells at the exponential growth phase using Lipofectamine 3000. Following a 24-hour transfection period, cells were seeded into Millicell EZ SLIDE (Merckmillipore) and treated with either FXT (15μM) or CQ (15μM) for 24 hours. After treatment, cells were fixed with 4% paraformaldehyde at room temperature for 30 minutes, washed with PBS, and stained with a DAPI solution (5 µg/ml) at 37°C for 5 minutes. Confocal microscopy was used to image and quantify the expression levels of GFP-RFP-LC3B in the transfected cells.

### 2.8 mRNA sequencing experimental method and analysis process

Total RNA was isolated employing the TRIzol reagent (Invitrogen, CA, USA) following the provided protocol. To ensure purity and quantify RNA, the NanoDrop 2000 spectrophotometer by Thermo Scientific in the USA was utilized. RNA integrity was evaluated through the Agilent 2100 Bioanalyzer (Agilent Technologies, Santa Clara, CA, USA). Subsequently, libraries were prepared using the VAHTS Universal V6 RNA-seq Library Prep Kit as per the provided guidelines. Transcriptome sequencing and analysis were executed by OE Biotech Co., Ltd. (Shanghai, China).

GO (Gene Ontology), KEGG pathway, Reactome, and WikiPathways enrichment analyses of DEGs were performed using R (v 3.2.0) based on the hypergeometric distribution to identify significantly enriched terms. Column diagrams, chord diagrams, and bubble diagrams were generated using R (v 3.2.0) to visualize the significant enrichment terms. Gene Set Enrichment Analysis (GSEA) was conducted using GSEA software, ranking genes by their differential expression degrees in the two sample types, and testing predefined gene set enrichment at the top or bottom of the ranking list.

### 2.9 Establishment of metastasis models

Metastasis models were established in C57BL/6 mice. Brain metastasis was induced by inoculating 7.5×10^3^ B16 Br-luc cells in 100 µL Hank's balanced salt solution (HBSS) into the internal carotid artery, and lung metastasis was induced by injecting 20×10^4^ B16-luc cells in 100 µL of sterile HBSS into the tail vein. Mice were divided into FXT treatment group or vehicle treatment group, and on the first or the third day post-inoculation, FXT was administered via intraperitoneal injection (i.p.) at a dosage of 30mg/kg/day, once daily. Metastasis growth was monitored by using imaging system (IVIS, Perkin Elmer) to measure the bioluminescence signal subsequent after i.p. injection of 15 mg/kg D-luciferin.

### 2.10 Analysis of immune cells in mouse models of melanoma lung metastasis

About 20 days post-establishment of experimental lung metastasis in mice, lung and spleen tissues were dissected into small pieces. Lung pieces underwent enzymatic digestion with collagenase at 37°C for 2 hours. Following digestion, both lung and spleen pieces were filtered through a 70-micron strainer to obtain a single-cell suspension. After erythrocyte lysis using red blood cell lysis buffer (BL503B, Biosharp, Hefei, China) at room temperature for 5 minutes, cells were stained with specific antibodies in the dark at 4°C for 30 minutes and subsequently analyzed via FMC using an ACEA NovoCyte instrument.

### 2.11 Statistical analysis

The means of the data from three independent experiments are presented, along with a measure of variability. The variability is expressed either as the standard deviation (S.D.) or the standard error of the mean (S.E.M.) and analyzed by GraphPad Prism 8. Two types of statistical tests are mentioned, depending on the nature of the data and assumptions: a. Two-tailed Student's t-tests: This test is commonly used when comparing two groups, assuming the data follows a normal distribution and has equal variances. b. Mann-Whitney U tests: This non-parametric test is used when the data do not meet the assumptions of normality or equal variances. The P-values obtained from the statistical tests are denoted with asterisks, indicating their significance levels. The significance levels commonly used in this notation are: *P < 0.05: Indicates a statistically significant difference between groups at the 5% significance level. ** P < 0.01: Indicates a statistically significant difference at the 1% significance level. *** P < 0.001: Indicates a highly statistically significant difference at the 0.1% significance level.

## 3. Results

### 3.1 FXT has an inhibitory effect on the growth of melanoma cells in vitro

**Figure 1:**
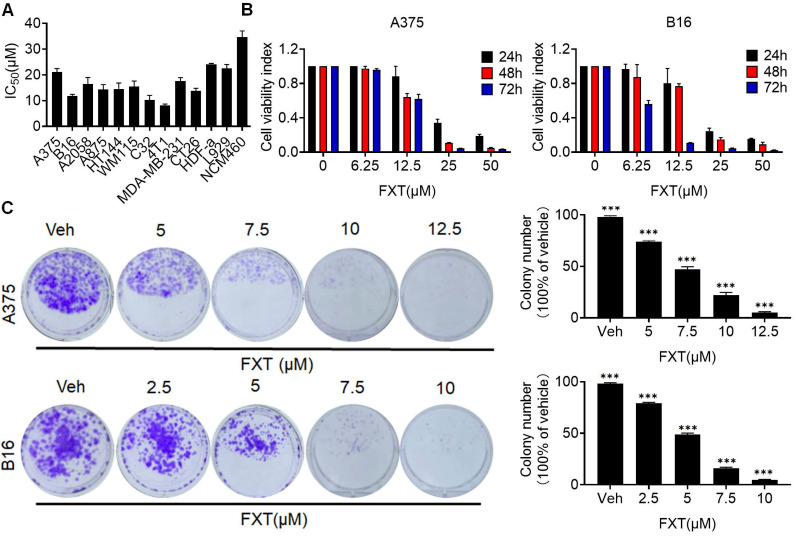
FXT has an inhibitory effect on the growth of melanoma cells in vitro. (A) IC50 Calculation: The IC50 values of FXT on various melanoma cell lines, other tumor cells and normal cells were determined using Prism 8 software. Each value represents the concentration of FXT required for 50% inhibition of cell growth. (B) Cell Viability Assay: A375 and B16 cells were subjected to varying doses of FXT for 24, 48, and 72 hours. Cell viability was assessed using the MTT assay, measuring the metabolic activity of the cells. (C) Colony Formation: Representative images of A375 and B16 cell colonies formed after treatment with different concentrations of FXT or without FXT for 7 days are shown. Quantitative results of colony formation are presented on the right side of the image. Data is presented as mean ± S.D. *p < 0.05, **p < 0.01, ***p < 0.001. Group comparisons were conducted using either two-tailed Student t-test or Mann-Whitney U test.

In this study, the potential therapeutic effect of FXT on melanoma was investigated. The findings indicated that FXT effectively inhibited the growth and proliferation of several melanoma cell lines. The half-maximal inhibitory concentration (IC50) values of FXT were in the low micromolar range, indicating its potency in inhibiting the growth of melanoma, colon cancer and breast cancer cells (Fig. 1A). Furthermore, FXT exhibits a lower inhibitory effect on normal cells compared to tumor cells. B16 represents a highly aggressive murine melanoma cell line, while A375 denotes a commonly employed malignant human melanoma cell line harboring the BRAFV600E mutation. Notably, the inhibitory effect was more pronounced with higher concentrations of FXT and longer treatment durations (Fig. 1B).

To further evaluate the long-term effects of FXT on melanoma cell growth, colony formation assays were performed. The results showed that FXT treatment at low micromolar concentrations significantly inhibited colony formation of melanoma cells (Fig. 1C, D). These discoveries offer initial indications or preliminary evidence of the potential of FXT to inhibit the growth of melanoma cells in vitro, suggesting its effectiveness in suppressing melanoma progression.

### 3.2 FXT exerts its therapeutic effects by inducing G0/G1 cell cycle arrest

**Figure 2:**
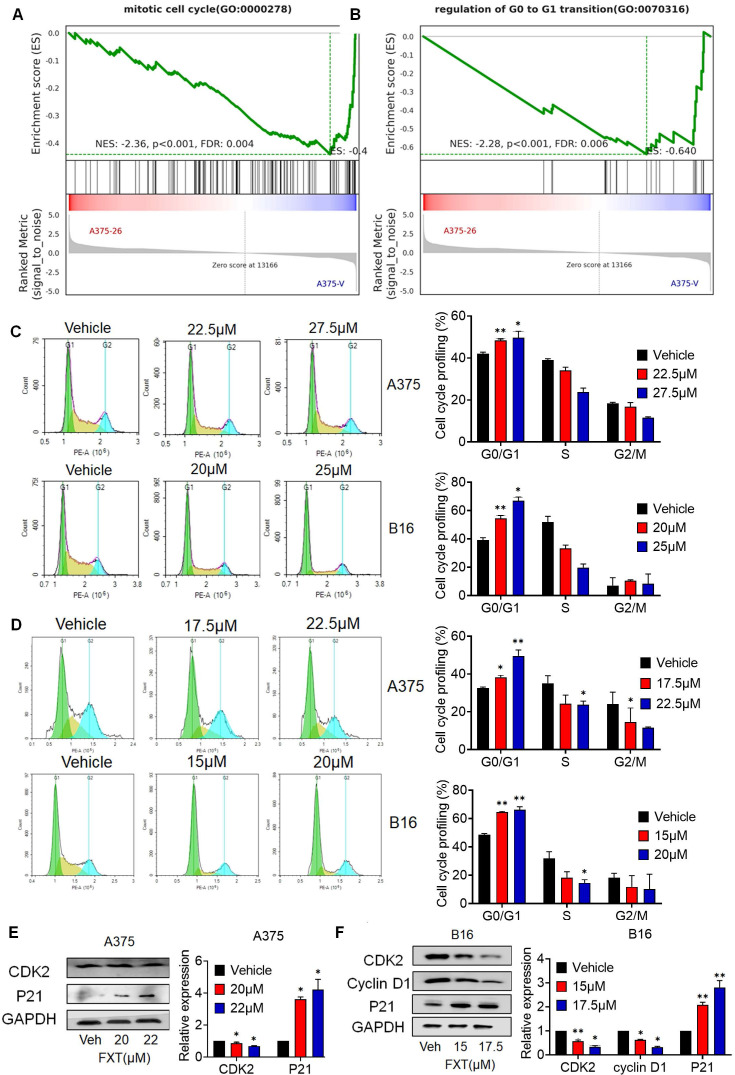
FXT exerts its therapeutic effects by inducing G0/G1 cell cycle arrest. (A, B) GSEA of DEGs. (C, D) A375 and B16 cells were treated with FXT for 12h and 24 h, followed by staining with PI and analysis using flow cytometry. Cell cycle phase distribution was determined based on DNA content and the percentages of cells in different cell cycle phases (G0/G1, S, G2/M) were quantified in A375 and B16 cells after FXT treatment. Data are derived from three independent experiments (n=3). (F, G) The expression levels of proteins related to G0/G1 cell cycle regulation were assessed in A375 (E) and B16 (F) cells after 48 hours of FXT treatment using Western blot analysis. Quantification of protein expression in A375 and B16 cells was performed. Data is presented as mean ± S.D. *p < 0.05, **p < 0.01, ***p < 0.001. Group comparisons were conducted using either two-tailed Student t-test or Mann-Whitney U test. CDK2: Cyclin-dependent kinase 2; P21: Cyclin-dependent kinase inhibitor 1A; GAPDH: glyceraldehyde-3-phosphate dehydrogenase.

Cell division and cell cycle control hold significant importance in cancer development, and the dysregulation of the cell cycle leads to abnormal cell proliferation, which is a common feature of human cancer 29, so targeting cell cycle control is an important strategy in cancer treatment. To investigate the mechanism by which FXT inhibits melanoma cell growth, we performed GSEA, which revealed a significant reduction in the arrest of the cell cycle, mitotic cell cycle, and regulation of G0 to G1 transition upon FXT treatment (Fig. 2A-B).

To confirm the effect of FXT on the cell cycle, FMC was conducted to evaluate the cell cycle distribution of melanoma cells after FXT treatment. The data demonstrated which FXT induced a significant cell cycle arrest at the G0/G1 phase in melanoma cells 12 hours and 24 hours after treatment. This finding suggests that FXT interferes with cell cycle progression by modulating cell cycle-related proteins, leading to G0/G1 phase arest and inhibiting melanoma cell growth (Fig. 2C, D).

We further examined the levels of key regulatory proteins implicated in the G0/G1 phase of the cell cycle were examined. Consistent with previous studies, FXT treatment resulted in a notable reduction in the expression of Cyclin-dependent kinase 2 (CDK2), a critical regulator of cell cycle progression, while the accumulation of P21, a cell cycle inhibitor and anti-proliferative factor, was significantly increased (Fig. 2E, F). Additionally, the levels of Cyclin D1, a cell cycle regulatory factor, were reduced in B16 cells following FXT treatment (Fig. 2F). These findings observations suggest that FXT exerts its therapeutic effects by affecting cell cycle-related proteins and inducing G0/G1 cell cycle arrest in melanoma cells.

### 3.3 FXT induces intrinsic apoptosis in melanoma cells

**Figure 3:**
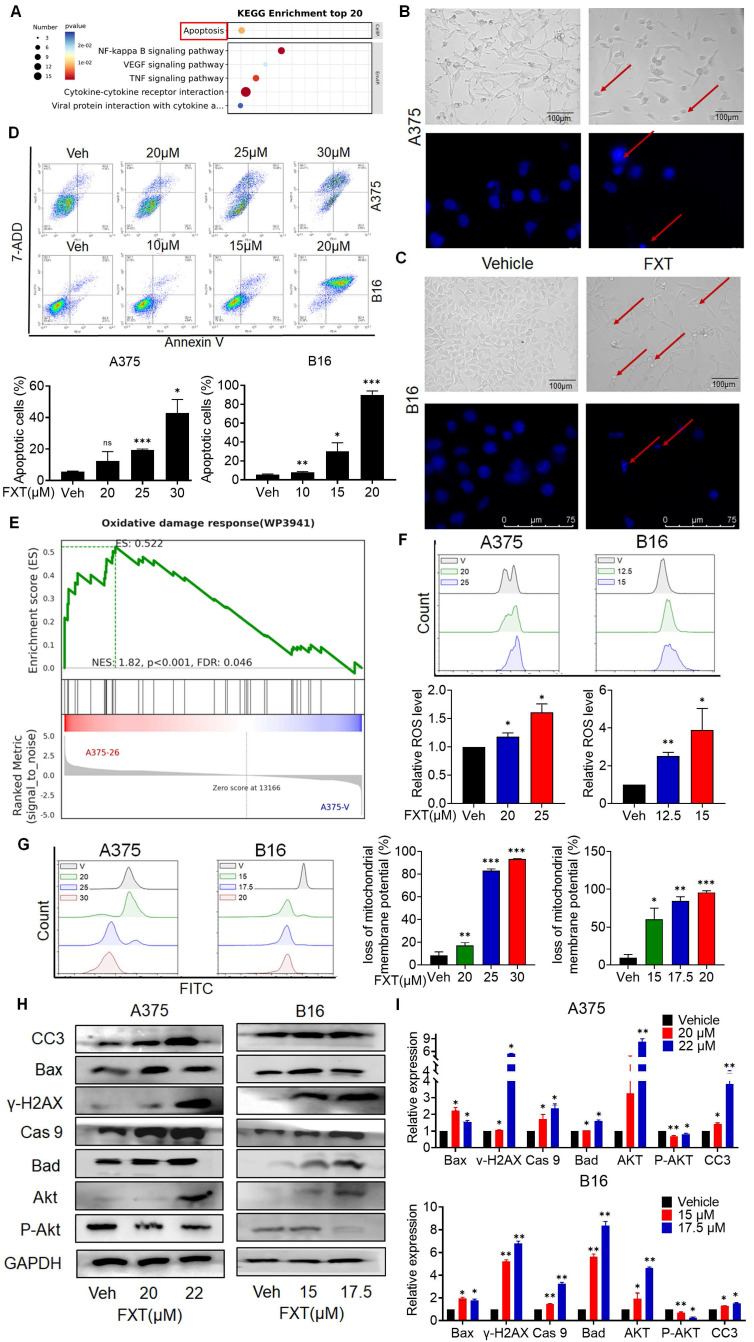
FXT induces intrinsic apoptosis in melanoma cells. (A) KEGG Pathway Analysis: A375 cells were treated with or without FXT (21μM) for 48 hours, and KEGG pathway analysis was conducted. (B, C) Upper Panels: The impact of FXT on melanoma cell morphology was assessed. A375 and B16 cells were exposed to corresponding concentrations of FXT for 48 hours. Following the treatment, bright-field microscope images were captured to visualize the cells. Scale bar: 100µm. Lower Panels: Nuclear changes in tumor cells post-treatment with FXT were examined. Cells were treated with FXT at specified concentrations for 24 hours and then stained with Hoechst 33342 (10 μg/ml) to observe nuclear morphology. Red arrows indicate nuclear fragmentation and bright-blue fluorescence highlighting condensed nuclei. Scale bar: 75µm. (D) Apoptosis Analysis: The effects of FXT on apoptosis in A375 and B16 cells were examined after 48 hours of treatment. Apoptosis was observed and quantified using Annexin V-PE and 7-AAD staining, followed by flow cytometry analysis. The data were collected from three independent experiments (n=3). (E) GSEA: GSEA was performed on A375 cells treated with or without FXT for 48 hours. The analysis identified enriched gene sets, providing insights into the molecular pathways and biological processes affected by FXT treatment. (F) Intracellular ROS Levels: Intracellular ROS levels in A375 and B16 cells were evaluated following 48 hours of FXT treatment. DCFH-DA staining combined with flow cytometry analysis allowed for the detection of intracellular ROS levels. Quantitative results, based on three independent experiments (n=3), are provided. (G) Mitochondrial Membrane Potential: The impact of FXT on mitochondrial membrane potential in A375 and B16 cells were evaluated after 48 hours of treatment. Rh123 staining was utilized, followed by flow cytometry analysis to measure changes in mitochondrial membrane potential. Quantitative results, derived from three independent experiments (n=3), are presented. (H, I) Expression of Apoptosis-Related Proteins: The expression levels of key apoptosis-related proteins in A375 and B16 cells treated with different concentrations of FXT for 48 hours were examined. Data is presented as mean ± S.D. *p < 0.05, **p < 0.01, ***p < 0.001. Group comparisons were conducted using either two-tailed Student t-test or Mann-Whitney U test. CC3: Cleaved Caspase 3; Bax: B- cell lymphoma -2-Associated X; γ-H2AX: H2A histone family member X; Cas 9: Caspase 9; Bad: BCL2 associated agonist of cell death; Akt: protein kinase B; GAPDH: glyceraldehyde-3-phosphate dehydrogenase.

To understand how FXT inhibits melanoma, RNA sequencing (RNA-seq) analysis was performed on FXT-treated A375 cells. Differential gene expression analysis revealed statistically significant genes (p < 0.05, log2 fold change > ±1), identifying 307 genes-273 upregulated and 34 downregulated genes following p-value correction. Using the DAVID bioinformatics tool and KEGG analysis, we categorized and examined these genes, uncovering several potential downstream signaling pathways influenced by FXT in treating melanoma. Notably, apoptosis emerged as a significant signaling pathway (Fig. 3A), suggesting that FXT might trigger apoptosis in melanoma cells.

The induction of apoptosis is a frequently observed mechanism of action for anticancer agents. In previous studies, FXT has been demonstrated to induce apoptosis in cancer cells. Our examination of melanoma cell lines post-FXT treatment revealed distinct signs of cell contraction, a common hallmark of apoptosis (Fig. 3B, C). Subsequent Hoechst 33342 staining conducted after 24 hours of FXT exposure displayed characteristics such as bright-blue fluorescence in condensed nuclei, reduced cell volume, and nuclear fragmentation, reinforcing the presence of apoptotic features (Fig. 3B, C). To validate this, Annexin V/7-AAD staining experiments were conducted, and the results showed a significant increase in both early and late apoptotic melanoma cells after 48 hours of FXT treatment (Fig. 3D). Furthermore, GSEA indicated a notable enrichment of the Oxidative damage response pathway in melanoma cells treated with FXT, indicating a potential role of FXT in regulating intracellular oxidative damage response and apoptosis (Fig. 3E), indicating that FXT may regulate the apoptosis process of melanoma cells by influencing intracellular oxidative damage response. Additionally, oxidative damage can promote the generation of ROS and lead to various cellular outcomes, including proliferation, growth arrest, senescence, necrosis, or apoptosis, depending on different cell types and conditions 30. Thus, we quantitatively measured the levels of ROS and mitochondrial membrane potential in melanoma cells after FXT treatment using FMC, and the results showed that FXT treatment resulted in a decrease in mitochondrial membrane potential and an increase in ROS compared to the control group (Fig. 3F, G). These findings further support the possibility of FXT inducing apoptosis in melanoma cells and suggest the involvement of the intrinsic apoptotic pathway (mitochondrial pathway). The decrease in mitochondrial membrane potential and the increase in ROS are typical features of the intrinsic apoptotic pathway, implying mitochondrial dysfunction and the potential release of apoptotic-related molecules. However, additional research is necessary to validate these findings and delve deeper into the specific mechanisms through which FXT regulates oxidative damage response and mitochondrial function in melanoma cell apoptosis.

Next, we explored the mechanism of cell apoptosis by examining the expression levels of apoptosis-related proteins. Since the effect of FXT is concentration-dependent within certain concentration ranges, we investigated the impact of FXT at different concentrations on the expression of intrinsic apoptosis-related proteins. After treating A375 and B16 cells with different concentrations of FXT for 48 hours, we analyzed the expression of key proteins related to cell apoptosis. The results showed that FXT significantly upregulated the expression of the DNA damage sensor γ-H2AX and Akt, while downregulating the phosphorylation of Akt (Fig. 3H, I). Furthermore, apoptosis is a regulated process controlled by the Bcl-2 protein family. The family comprises antiapoptotic proteins like Bcl-2, along with two groups of proapoptotic proteins: effectors (e.g., BAX) and BH3-only proteins (e.g., BAD) 31. Additionally, the cleaved Caspase 3, responsible for morphological and biochemical changes in apoptosis, was also examined 32. Our data showed that FXT induced an increase in the expression of BAX, Bad, and caspase-9 in melanoma cells, which are key events in the process of cell apoptosis. In summary, these results suggest that FXT-induced cell death largely involves the mitochondria-mediated intrinsic apoptotic pathway. FXT promotes the apoptosis process in melanoma cells by influencing the expression of key proteins such as γ-H2AX, Akt, BAX, Bad, and caspase-9. These findings further support previous observations that FXT may induce melanoma cell apoptosis by modulating intracellular apoptotic signaling pathways and protein expression.

Collectively, the results suggest that FXT exerts its inhibitory effect on melanoma cells by modulating intracellular apoptotic signaling pathways and protein expression, highlighting the potential of FXT as a therapeutic agent for melanoma treatment.

### 3.4 FXT induces cell-protective autophagy and obstructs the flux of autophagic activity in melanoma cells

**Figure 4:**
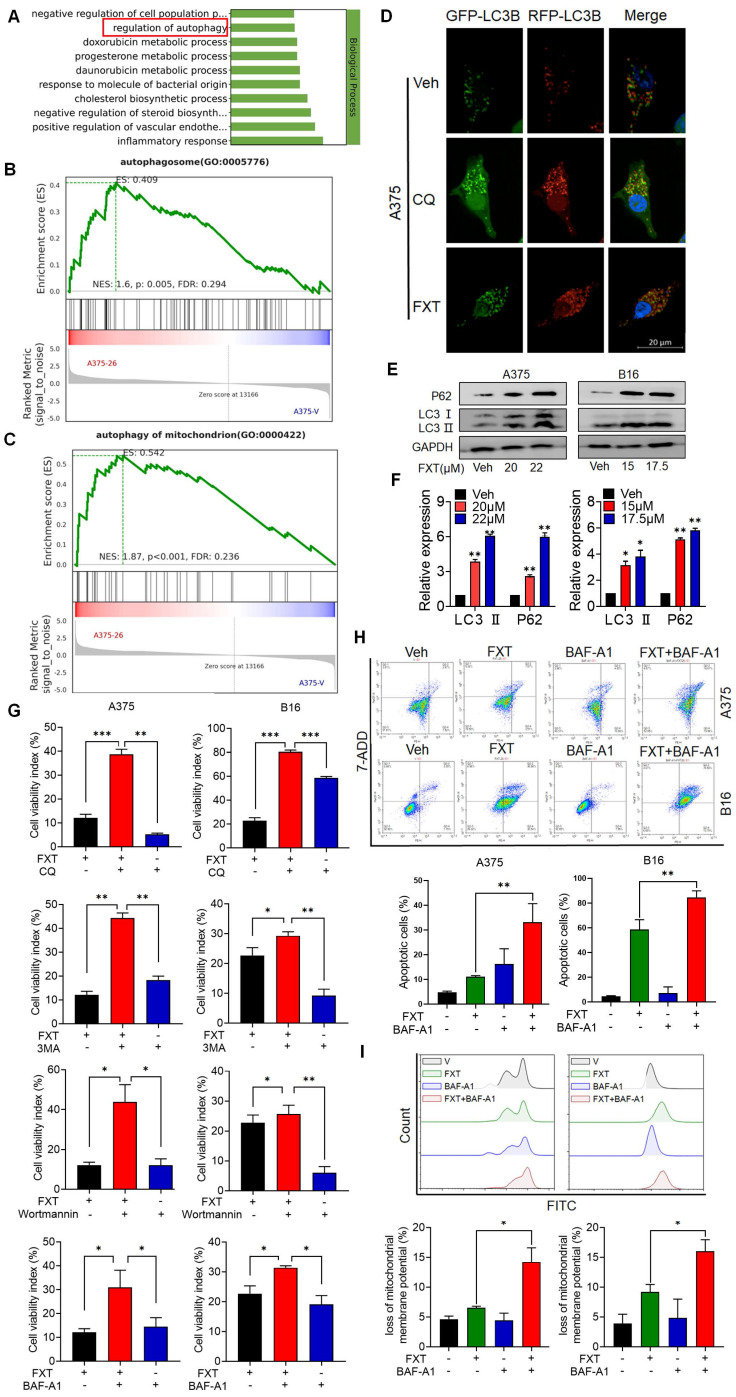
FXT induces cell-protective autophagy and blocks autophagic flux in melanoma. (A) GO analysis of DEGs. (B, C) GSEA of DEGs. (D) Representative images of A375 cells transfected with tandem RFP-GFP-labeled LC3B plasmid after treatment with FXT (15μM). Scale bar, 20µm. (E) Western blot analysis of LC3 and P62 expression levels in A375 and B16 cells after 48 hours of FXT treatment. (F) Quantification of LC3 and P62 expression levels in A375 and B16 cells after 48 hours of FXT treatment. (G) MTT assay of A375 cells treated with specified concentrations of FXT for 48 hours in the presence or absence of CQ (12.5μM), 3-MA (12.5μM), BAF-A1 (12.5μM), and Wortmannin (12.5μM). MTT assay of B16 cells treated with specified concentrations of FXT for 48 hours in the presence or absence of CQ (6.25μM), 3-MA (6.25μM), BAF-A1 (6.25μM), and Wortmannin (6.25μM). (H) Effects of BAF-A1 on FXT-induced apoptosis in A375 and B16 cells. A375 or B16 cells were treated with FXT alone or in combination with BAF-A1 for 48 hours, and cell apoptosis was detected using Annexin V-PE and 7-AAD staining, and flow cytometry results are shown (n=3). (I) Intracellular ROS levels were measured in A375 and B16 cells using DCFH-DA staining followed by flow cytometry analysis. The quantitative results, based on three independent experiments (n=3), are displayed. Data is presented as mean ± S.D. *p < 0.05, **p < 0.01, ***p < 0.001. Group comparisons were conducted using either two-tailed Student t-test or Mann-Whitney U test. P62: Sequestosome 1; LC3 II: light chain 3 II.

To gain insights into the mechanisms underlying the effects of FXT on melanoma cells, a re-analysis of the RNA-seq data was performed. GO analysis revealed that FXT treatment induced the regulation of autophagy (Fig. 4A), suggesting its involvement in the cellular response to FXT treatment. Autophagy is a cellular process involved in the degradation and recycling of cellular components, and its role in tumor development is complex. Currently, it is widely believed that autophagy inhibits tumor development. However, there is evidence suggesting that autophagy plays an important role in metabolic adaptation of tumor cells (e.g., by clearing dysfunctional mitochondria) and evasion of immune surveillance. Established tumors require autophagy to support uncontrolled cell growth and increased metabolic activity, leading to tumor dependency on autophagy 33.

GSEA results indicated a significant enrichment of autophagosome and autophagy of mitochondrion after FXT treatment (Fig. 4B, C). The formation of autophagosomes is a distinctive feature of the autophagic process, signifying the core mechanism of engulfing autophagic cargo 34. Autophagy can be broadly classified into non-selective autophagy and selective autophagy, depending on the way targets are sequestered for degradation. In selective autophagy, specific proteins called "autophagy receptors" recognize certain molecules, structures, or organelles and actively engulf them into autophagosomes 35. Cells employ different types of autophagy to maintain homeostasis or regulate their functions in response to different conditions 36. Mitophagy, the selective autophagy of mitochondria, plays an important role in maintaining mitochondrial homeostasis and is a critical mechanism for mitochondrial quality control. It has been shown to have both tumor-suppressive and tumor-promoting effects depending on the stage of tumor development 37, 38.

To investigate the role of autophagy in FXT-induced melanoma cell death, several assays were conducted. Microtubule-associated protein light chain 3 (LC3) levels are closely correlated with the number of autophagosomes and are widely used as a marker for monitoring autophagy 39. p62 can bind to LC3 and is used as a selective substrate for monitoring autophagic flux 40.

Therefore, we analyzed the levels of LC3 conversion and p62 expression by immunoblotting and found that both LC3 and p62 levels were increased in melanoma cells after FXT treatment (Fig. 4E, F), indicating the induction of cellular autophagy. To explore the potential impairment in autophagic flux linked to failure in autophagosome-lysosome fusion, we transfected A375 cells with tandem RFP-GFP-tagged LC3B plasmids. Using chloroquine (CQ) as a positive control to inhibit autolysosomes, we observed the fluorescence puncta of LC3B. Interestingly, following FXT treatment, a majority of LC3B fluorescence puncta exhibited yellow dots (RFP+GFP+ signals), indicating the presence of autophagosomes, rather than red dots (RFP+GFP- signals), indicative of autolysosomes. This suggests that FXT treatment led to autophagosome accumulation and a reduction in autolysosome formation.

To further elucidate the role of autophagy in the anti-melanoma effects of FXT, we co-treated A375 and B16 cells with FXT and four commonly used autophagy inhibitors: early autophagy inhibitors chloroquine (CQ, an auto-phagolysosome fusion inhibitor) and Bafilomycin A1 (BAF-A1, a proton pump inhibitor), and late autophagy inhibitors Wortmannin (a PI3K inhibitor that blocks autophagy initiation and autophagosome formation) and 3-methyladenine (3-MA, a Class III PI3K inhibitor). Our data clearly showed that co-treatment of FXT with autophagy inhibitors significantly enhanced cell growth inhibition (Fig. 3G). Similarly, FMC results indicated that the combination of FXT and BAF-A1 synergistically induced apoptosis and increased ROS in tumor cells (Fig. 4H, I).

Collectively, these findings indicate that FXT induces cell-protective autophagy and apoptosis, while blocking autophagic flux. The disruption of autophagic flux enhances the anti-melanoma effects of FXT, suggesting a potential therapeutic strategy for melanoma treatment.

### 3.5 FXT effectively inhibits the growth of melanoma in mouse models of lung and brain metastasis

**Figure 5:**
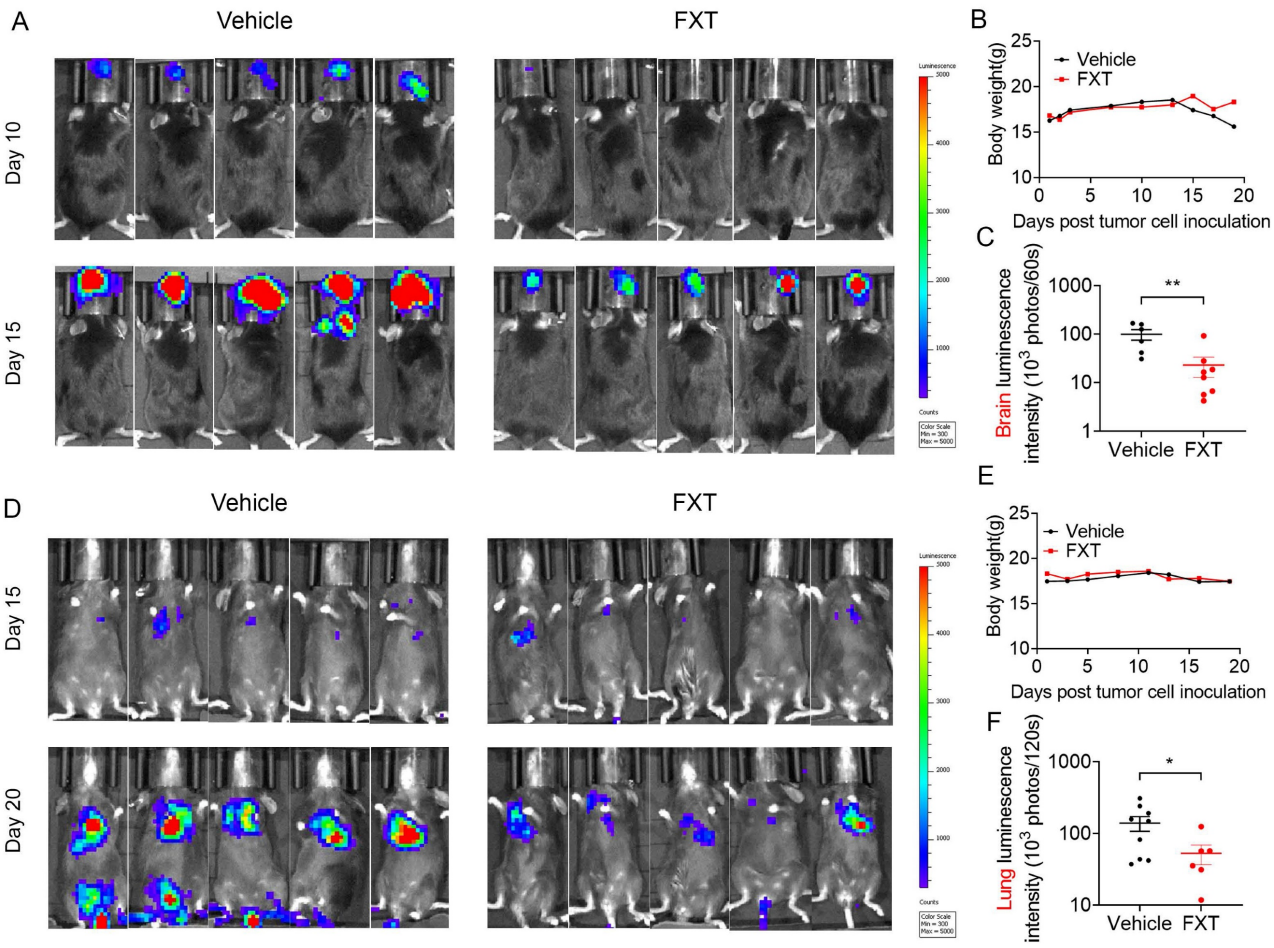
FXT effectively inhibited melanoma growth in mice in lung metastasis and brain metastasis models. (A) Displaying representative in vivo luminescence images capturing brain metastasis signals from each treatment group at day 10 and 15 post-tumor cell inoculation. (B) Observing and recording the body weights of mice across different treatments in the B16 brain metastasis model (n = 8). (C) Quantifying luminescence intensities specifically in brain areas at day 15 after varied treatments (n = 6-8). (D) Presenting representative in vivo luminescence images showcasing lung metastasis signals from each group at day 15 and 20 following tumor cell inoculation. (E) Observing the body weights of mice exposed to different treatments in the B16 lung metastasis model (n = 9). (F) Quantifying luminescence intensities in lung areas at day 20 after diverse treatments (n = 6-9). Data is presented as mean ± S.D. *p < 0.05, **p < 0.01, ***p < 0.001. Group comparisons were conducted using either two-tailed Student t-test or Mann-Whitney U test.

Metastatic melanoma is an aggressive disease with a 5-year survival rate of 16% and poor response to most standard chemotherapies 41. Previous studies, including our own research, have shown that many antipsychotic drugs can cross the BBB and have potential anticancer effects 42, 43. To evaluate the inhibitory effects of FXT on melanoma metastasis, particularly brain metastasis and lung metastasis, mouse models were established. In the mouse model of brain metastasis, B16 cells were injected into the carotid artery of C57 mice, and daily treatment with FXT at a dose of 30 mg/kg was initiated on the second day after inoculation (Fig. 5A-C). The results demonstrated that FXT strongly inhibited the growth of mouse melanoma brain metastasis without causing significant changes in the body weight of the mice.

Similarly, in the mouse model of experimental lung metastasis, B16 cells were injected into the tail vein of C57 mice, and daily treatment with FXT at a dose of 30 mg/kg was initiated on the third day after inoculation. FXT treatment significantly inhibited the growth of mouse melanoma lung metastasis, while no significant changes in body weight were observed (Fig. 5D-F).

These findings highlight the effectiveness of FXT in suppressing melanoma metastasis, particularly brain metastasis and lung metastasis, in preclinical mouse models. This provides valuable insights for the treatment of metastatic melanoma, especially in patients who have poor responses to standard chemotherapies.

### 3.6 FXT regulates the population of important immune cells in the mouse model of melanoma lung metastasis

**Figure 6:**
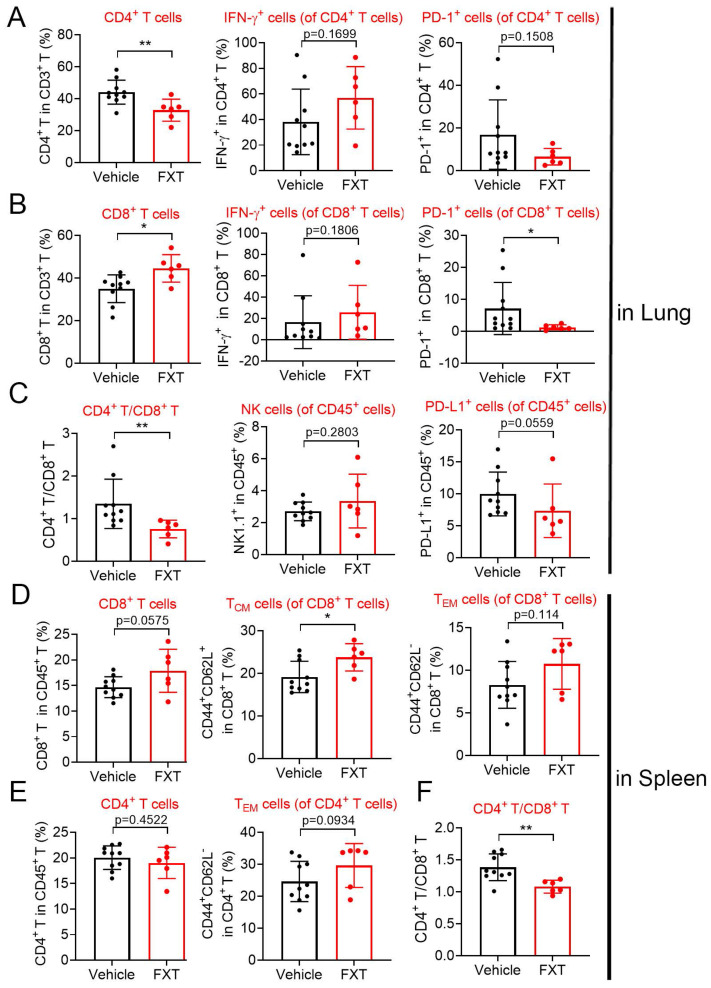
The effects of FXT on the infiltration of some immune cells in the lung and spleen microenvironment. The lung and spleen tissues of the mice were collected to prepare sing cell suspensions. The cells were then stained with different sets of fluorescein-conjugated antibody and analyzed with flow cytometry to determine the frequencies of some important cells involved in anticancer immunity. (A-C) The percentage of CD4^+^ T cells, IFN-γ^+^ CD4^+^ T-cells, PD-1^+^ CD4^+^ T cells, CD8^+^ T cells, IFN-γ^+^ CD8^+^ T-cells, PD-1^+^ CD8^+^ T cells, CD4^+^/CD8^+^, NK cells and PD-1^+^ CD45^+^ T cells within the leukocyte population in the lung (n=6-10). (D-F) The percentage of CD8^+^ T cells, T_CM_ cells of CD8^+^ T cells, T_EM_ cells of CD8^+^ T cells, CD4^+^ T cells, T_EM_ cells of CD4^+^ T cells and CD4^+^/CD8^+^ within the leukocyte population in the spleen. Data is presented as mean ± S.D. *p < 0.05, **p < 0.01, ***p < 0.001. Group comparisons were conducted using either two-tailed Student t-test or Mann-Whitney U test.

Indeed, the tumor microenvironment (TME) significantly influences tumor advancement and the effectiveness of treatment responses. To investigate the impact of FXT on the immune environment, we utilized a mouse model of experimental lung metastasis by injecting B16 cells into the tail vein of C57 mice. After 20 days of FXT treatment, we examined the infiltration of immune cells in the lung tumor microenvironment. The results revealed certain changes in the immune cell population, including CD3^+^ cell, interferon-gamma (IFN-γ) ^+^ CD8^+^ T-cells, programmed death-1 (PD1) ^+^ CD4^+^ T-cells, IFN-γ^+^ CD4^+^ T-cells, Nature kill cells, PD1^+^ CD45^+^ T-cells (Fig. 6A-C).

Interestingly, FXT treatment led to a significant increase in CD8^+^ T-cells (Fig. 6B), which have a direct role in eliminating cancer cells by recognizing and eliminating tumor cells. CD8^+^ T-cells have the ability to directly eliminate cancer cells and are responsible for recognizing and eliminating tumor cells. Their mechanisms of tumor cell killing include the release of IFN-γ and other cytokines, which play a very important role in anti-tumor immune responses 44 Furthermore, there was a significant decrease in PD1^+^ CD8^+^ T-cells (Fig. 6B), which are associated with tumor immune evasion 45.

In the spleen tumor microenvironment, although there were some changes in the population of CD4^+^ T-cells, CD8^+^ T-cells, effector memory T cells (T_EM_ s) in CD4^+^ T-cells and CD8^+^ T-cells, these alterations did not demonstrate statistical significance (Fig. 6D-F). Nonetheless, a notable or statistically significant increase in central memory T cells (T_CM_ s) in CD8^+^ T-cells (Fig. 6D). T_CM_ s can produce high levels of cytokines and exhibit strong cytotoxic activity in vitro, and they have been shown to have strong tumor eradication capabilities in mice 46.

The substantial rise in CD8^+^ T-cells and T_CM_ CD8^+^ T-cells, coupled with the decrease in PD1^+^ CD8^+^ T-cells, indicates that FXT has a substantial impact on the anti-tumor immune response. FXT promotes the establishment of an anti-cancer environment within the tumor microenvironment, which helps inhibit the growth of melanoma cells during the process of lung metastasis.

These discoveries underscore the potential of FXT as an immunomodulatory agent in the treatment of melanoma, a lethal form of cancer. FXT's ability to regulate the population of immune cells, particularly CD8^+^ T-cells, provides new insights into its therapeutic effects and suggests its potential as a valuable addition to current melanoma treatment strategies.

## 4. Discussion

Melanoma, originating from melanocytes, is a malignant tumor that predominantly occurs in the skin, eyes, and other mucosal tissues 47. The incidence of this disease is progressively increasing and represents a malignancy associated with relatively high mortality rates among other malignant tumors. Clinical management of melanoma encounters various challenges. Firstly, melanoma exhibits a high propensity for metastasis, and treatment strategies differ between early-stage and advanced-stage metastasis, necessitating comprehensive consideration of disease progression and individual patient variations 48. Secondly, traditional treatment modalities such as surgical resection, radiation therapy, and chemotherapy demonstrate limited effectiveness in the treatment of melanoma, emphasizing the urgent need for the development of rational and safe novel therapeutic approaches. Several new therapeutic strategies have gained significant attention in the treatment of melanoma, including immunotherapy and targeted therapy. These novel treatment modalities have exhibited notable efficacy in certain patients, although they still encounter limitations and challenges, such as drug resistance and immune-related toxicities.

Therefore, the development of more effective and safe treatment approaches for melanoma is urgently needed. FXT, a widely used antidepressant medication, has been extensively applied in clinical practice for the treatment of depression 49. Its safety profile has been thoroughly validated, making it one of the primary drugs for depression treatment. FXT also demonstrates a high level of maturity in pharmaceutical manufacturing processes. Pharmaceutical companies have established standardized production processes and quality management systems to ensure the consistency and safety of FXT.

Moreover, several studies have indicated the potential of FXT in inhibiting tumor growth 20, 21, 50. In previous research, FXT has been found to exhibit inhibitory effects on various types of tumor cells 20, 21, 50. These inhibitory effects may involve multiple mechanisms, including the modulation of cell proliferation 20, 21, induction of apoptosis 23, and suppression of tumor angiogenesis 25. However, it should be noted that despite the promising inhibitory effects of FXT on tumors observed in these studies, its application in clinical tumor treatment is still in the preliminary stage.

Apoptosis is a programmed cell death process that is considered one of the important mechanisms by which anti-tumor drugs exert their effects 51. It involves the regulation of intracellular molecular signaling pathways, leading to characteristic changes such as DNA fragmentation, nuclear condensation, and membrane blebbing, ultimately resulting in cell death 52. We found that in melanoma, FXT can induce mitochondria-mediated intrinsic apoptosis. Mitochondria play a crucial role in cell apoptosis by releasing cytochrome c, activating caspase family proteins, and modulating the progression of apoptosis 53. Our research revealed that FXT can impact mitochondrial function in melanoma cells, leading to mitochondrial membrane depolarization, increased ROS, and caspase activation, thereby inducing apoptosis in melanoma cells. The discovery of this FXT-induced intrinsic apoptosis mechanism in melanoma cells provides a scientific basis for its potential application as an anti-tumor drug. However, it should be noted that while FXT has demonstrated apoptotic induction effects on melanoma cells, its application in the clinical treatment of melanoma still requires further research and validation.

The cell cycle and cell apoptosis are closely related cellular physiological processes 54. The cell cycle refers to the orderly progression of cells through a series of phases during growth and reproduction, including the G1 phase (growth phase), S phase (DNA synthesis phase), G2 phase (pre-mitotic phase), and M phase (mitotic phase) 55. The regulation of the cell cycle is influenced by various intrinsic and extrinsic factors, including cell cycle proteins (Cyclins), cyclin-dependent kinases (CDKs), and cell cycle inhibitors 56. In our research, we found that FXT may affect the cell cycle regulation in melanoma cells, resulting in cell arrest at the G0/G1 phase, hindering entry into the S phase and further proliferation. This cell cycle arrest may be associated with the therapeutic effect of FXT on melanoma cells. Additionally, there is a connection between cell cycle regulation and cell apoptosis. During the cell cycle process, if cells detect DNA damage or other abnormalities, they may pause at specific cell cycle stages to allow for DNA repair or regulation 54. If the DNA damage cannot be repaired, cells may initiate the apoptotic pathway to induce programmed cell death 54. Therefore, cell cycle arrest and cell apoptosis can influence each other and play important roles in cellular activities.

Autophagy is a cellular self-degradation process that plays a critical role in maintaining cellular homeostasis, clearing abnormal proteins and organelles, and responding to environmental stress 57. Autophagy involves the sequestration of specific cellular components into double-membrane vesicles called autophagosomes, which are then delivered to lysosomes for degradation and recycling 58. Key regulatory factors in the process of autophagy include autophagy-related genes (ATGs) and signaling pathways such as mTOR (mammalian target of rapamycin) and AMPK (5'-AMP-activated protein kinase) 59. Autophagy is generally considered a cellular protective response that provides energy and building blocks to sustain cell survival under stress or damage conditions 60. Our research has found that FXT induces cytoprotective autophagy in melanoma and inhibits autophagic flux. This suggests that FXT may trigger autophagy in melanoma cells as a response to stress and damage. However, blocking autophagic flux can lead to the accumulation of autophagosomes and impaired function, thereby interfering with normal autophagy processes in cells. These findings provide important insights for further investigating the mechanisms of action of FXT in melanoma treatment.

Metastasis is a significant challenge in anti-tumor research, as the majority of cancer-related deaths are attributed to tumor metastasis 61. Metastasis refers to the invasion of tumor cells from the primary site into surrounding tissues or their migration to distant organs or tissues through the circulatory system 62. This process involves multiple complex steps, including tumor cell invasion, intravasation or lymphatic vessel invasion, dissemination through the bloodstream or lymphatic system, adhesion, and invasion into new tissues 62. Brain metastasis and lung metastasis are among the primary modes of metastasis for common malignancies. Brain metastasis refers to the spread of tumor cells to brain tissue, forming metastatic lesions in the brain, while lung metastasis refers to the migration of tumor cells to the lungs, forming metastatic foci. The formation of these metastatic lesions significantly impacts patient prognosis and treatment outcomes. Overcoming brain and lung metastasis is a critical goal in anti-melanoma research. FXT has been discovered to exhibit inhibitory effects against melanoma in numerous prior studies. However, earlier research predominantly concentrated on its influence on tumor growth. Presently, our attention is turning towards probing its effects on melanoma metastasis, particularly concerning vital organs such as the brain and lungs. This line of inquiry bears the promise of introducing novel avenues for prolonging patient survival. Our study indicates that FXT exhibits anti-melanoma effects in *in vivo* models of brain and lung metastasis. Additionally, we found that FXT may exert its anti-melanoma lung metastasis effect through influencing the tumor microenvironment, among other factors.

We conducted an analysis of the immune microenvironment in the lung tissues and spleen tissues of mice in a lung metastasis model. Our study revealed significant alterations in the immune microenvironment of the lung metastasis model. The quantity of CD8^+^ T cells in the lung tissues was significantly upregulated, while the number of PD-1^+^ cells within these CD8^+^ T cells was significantly downregulated. Concurrently, the number of T_CM_ cells within CD8^+^ T cells in the spleen was significantly upregulated. These significant changes have a positive impact on the immune microenvironment. CD8^+^ T cells are essential immune cells that directly recognize and eliminate tumor cells 63. Thus, the upregulation of CD8^+^ T cells in lung tissues may contribute to enhancing the immune response against tumor cells. Additionally, PD-1 is an immune checkpoint protein, and its overexpression can lead to immune tolerance and tumor escape 64. Therefore, the downregulation of PD-1^+^ cell numbers in lung tissues may help restore the anti-tumor function of CD8^+^ T cells. In the spleen, the upregulation of T_CM_ cell numbers within CD8^+^ T cells indicates the establishment of immune memory. T_CM_ cells are memory immune cells with long-term survival and reactivation potential. They can maintain long-lasting immune memory against tumors within the body and rapidly initiate an immune response when needed 65. Our results revealed significant alterations in the population of immune cells, including CD8^+^ T cells, PD1^+^ CD8^+^ T cells, and T_CM_ CD8^+^ T cells. These changes suggest a positive impact on the immune response against tumors and the establishment of immune memory. FXT may modulate the immune system to enhance the anti-tumor immune response, although further research is needed to elucidate the underlying mechanisms.

Our research offers initial indications of FXT's potential in treating melanoma brain metastases and lung metastases. However, these findings necessitate further confirmation in models more closely resembling clinical conditions, such as testing with patient-derived tissues and cells. Regrettably, due to our current lack of access to such samples, these investigations have not been pursued. The clinical effectiveness of FXT hydrochloride in treating melanoma brain metastases and lung metastases still awaits validation through clinical trials. It is only through these trials that we can ascertain whether FXT could emerge as an efficacious treatment for melanoma, providing renewed hope for patients afflicted with the condition.

## 5. Conclusions

Our study highlights the potential of FXT as a promising treatment for melanoma. FXT exerts its anti-melanoma effects through multiple mechanisms, including apoptosis induction, autophagy modulation, cell cycle arrest, and inhibition of metastasis. Moreover, FXT may influence the immune microenvironment, promoting anti-tumor immune responses. These findings offer significant insights into the development of FXT as a novel treatment approach for melanoma. However, additional preclinical and clinical studies are warranted to further investigate its efficacy, safety, and potential combination with existing therapies to improve patient outcomes.
